# Medical News and Miscellany

**Published:** 1889-07

**Authors:** 


### Medical News and Miscellany.

**In** writing to advertisers, don't fail to mention the JOURNAL.

**Subscribers** are requested to promptly renew their subscriptions. In writing to our advertisers, give us credit.

**New York Polyclinic.**—Dr. C. Coe has been elected to the professorship made vacant by the death of Professor James B. Hunter.

**If any** of our readers would like to know something of Sulfonal, they will find the second cover page of this issue very interesting reading.

**In** next issue there will be an account of a most extraordinary case of elephantiasis, with an engraving of the subject, from the practice of Dr. Bennett.

**The Board of Dental Examiners,** provided for by a recent act of the Texas Legislature, to regulate the practice of dentistry in Texas, consists of Drs. Styles and LeSeur, of Austin, and Dr. Bowden, of Round Rock.

**Such is Fame.**—The newspapers, in reporting a case of railroad injury, in which Dr. W. A. Acheson, of Denison, was called, got his name "W. A. Chesen" and "W. Ahessen." There is no better known physician in the State, and the newspapers ought to be ashamed of this distortion.

**We want** to suggest to delinquent members of the State Association that the Transactions will be ready for distribution next month, and if they would expect a copy they should remit to Dr. Larendon at least a year's dues, if they cannot pay in full. This is but fair, and we give timely notice. Should any gentleman, knowing that he is in arrears for his dues, fail to receive a copy, he must not blame the Secretary nor the Publishing Committee. Orders must be obeyed.

---

**Dr. T. J. Jordan,** of Ledbetter, was killed at that place on the 11th inst., by a Mr. Lon Hill, of Manor. Particulars not learned. Dr. Jordan was a recent graduate of Louisville Medical College and we understand came to Texas from Nashville, where his parents reside. The remains were shipped to Nashville.

---

**We** are collecting for the August number, the views and opinions of members on the banquet question, together with the reasons for such views. "Banquet or no banquet" at Ft. Worth. Members will please drop us a line, expressive of their ideas as to the banquet as a feature of our annual meetings. We have some good ones on file.

---

**Dr. R. T. Knox,** ex-First Vice-President Texas State Medical Association, we regret to learn, has been, and is, quite seriously ill, at his home in Gonzales. The doctor is suffering with malarial dysentery, and although sick for some time back, he kept going his rounds, until too weak to ride, when he was compelled to take to his bed. We sincerely trust he may soon recover, and his many patients enjoy, as before, the benefit of his skill and kind attention.

---

**The Texas Pharmacy Board.**—The Governor has appointed Messrs. W. L. Mann, of Georgetown, R. E. Stromberg and T. M. Andrews, of Austin, to constitute the Board of Examiners provided for under the Pharmacy act, (which act was published in full in our June issue). The board will meet, organize, and elect a Registrar, and will hold its first session for the examination of druggists on August 12th proximo, in Austin.

---

**Rare Opportunity**—To buy a snug and beautiful home and good practice, with a forty acre farm and orchard, with everything ready to go to housekeeping and practice. Situated in a valley where perpetual spring prevails, sixty-five miles from San Antonio, and is just the place for an invalid physician, as a residence there has cured consumption. Enquire for particulars of the editor of this journal. Terms reasonable.

---

**Dr. R. L. Grant,** lately with a St. Louis firm, is now traveling in Texas in the interest of that stannch and reliable manufacturing house, REED & CARNRICK, of 147 Greenwich street, New York, a house well known to, and appreciated by the Texas profession. We congratulate Messrs. Reed & Carnrick in securing the services of such a pushing, competent, experienced and reliable man as Dr. Grant, and we commend the doctor and the house he represents, to our readers;—they'll "do to tie to."

---

**From** the Houston Post we learn that Dr. E. T. Cook, a well known physician of Trinity, has removed to Houston, and is the appointee elect to the position of Surgeon of the Southern Pacific and San Antonio and Aransas Pass Railroads, with headquarters at Houston. Dr. Cook is not only an efficient and successful physician and surgeon, but a genial and good citizen, worthy of a universal welcome to Houston.

---

**One Subscriber,** in renewing this month, says: "Like wine and a meerschaum pipe, the JOURNAL improves with age." All are cordial in the expression of their high appreciation of the JOURNAL, and well wishes for its continued success. There is no doubt of its continued boom. See the display of ads. in this issue, as an indication of its popularity, and appreciation of its value.

